# Two Domains of Vimentin Are Expressed on the Surface of Lymph Node, Bone and Brain Metastatic Prostate Cancer Lines along with the Putative Stem Cell Marker Proteins CD44 and CD133

**DOI:** 10.3390/cancers3032870

**Published:** 2011-07-13

**Authors:** Nicole F. Steinmetz, Jochen Maurer, Huiming Sheng, Armand Bensussan, Igor Maricic, Vipin Kumar, Todd A. Braciak

**Affiliations:** 1 Case Western Reserve University, Department of Biomedical Engineering, 10900 Euclid Ave, Cleveland, OH 44106, USA; E-Mail: nicole.steinmetz@case.edu; 2 Sanford-Burnham, Medical Research Institute, 10901 North Torrey Pines Road, La Jolla, CA 92037, USA; E-Mail: jmaurer@sanfordburnham.org; 3 Torrey Pines Institute for Molecular Studies, Division of Immune Regulation, 3550 General Atomics Court, San Diego, CA 92121, USA; E-Mail: hsheng@tpims.org (H.S.); 4 INSERM U976, Hôpital Saint Louis, F-75475 Paris, France; E-Mail: armand.bensussan@inserm.fr; 5 Department of Immunology, Dermatology and Oncology, Univ Paris Diderot, Sorbonne Paris Cité, UMRS976 F-75475 Paris, France; 6 Torrey Pines Institute for Molecular Studies, Laboratory of Autoimmunity, 3550 General Atomics Court, San Diego, CA 92121, USA; E-Mails: imaricic@tpims.org (I.M.); vkumar@tpims.org (V.K.)

**Keywords:** vimentin, prostate cancer, metastases, stem cell, Cowpea mosaic virus

## Abstract

Vimentin was originally identified as an intermediate filament protein present only as an intracellular component in many cell types. However, this protein has now been detected on the surface of a number of different cancer cell types in a punctate distribution pattern. Increased vimentin expression has been indicated as an important step in epithelial-mesenchymal transition (EMT) required for the metastasis of prostate cancer. Here, using two vimentin-specific monoclonal antibodies (SC5 and V9 directed against the coil one rod domain and the *C*-terminus of the vimentin protein, respectively), we examined whether either of these domains would be displayed on the surface of three commonly studied prostate cancer cell lines isolated from different sites of metastases. Confocal analysis of LNCaP, PC3 and DU145 prostate cancer cell lines (derived from lymph node, bone or brain prostate metastases, respectively) demonstrated that both domains of vimentin are present on the surface of these metastatic cancer cell types. In addition, flow cytometric analysis revealed that vimentin expression was readily detected along with CD44 expression but only a small subpopulation of prostate cancer cells expressed vimentin and the putative stem cell marker CD133 along with CD44. Finally, Cowpea mosaic virus (CPMV) nanoparticles that target vimentin could bind and internalize into tested prostate cancer cell lines. These results demonstrate that at least two domains of vimentin are present on the surface of metastatic prostate cancer cells and suggest that vimentin could provide a useful target for nanoparticle- or antibody- cancer therapeutic agents directed against highly invasive cancer and/or stem cells.

## 1. Introduction

Prostate cancer most frequently develops in men over the age of fifty and is responsible for the second greatest number of deaths in this age group second only to lung cancer [1]. When prostate cancers are found early enough while still localized within the prostatic capsule, the disease can be cured by radical prostactectomy [2]. However if this cancer has metastasized beyond the local prostate gland, no curative therapy currently exists and the disease can be lethal [3]. Under these conditions, death typically occurs due to the preferential abilities of certain cancer cells to metastasize to lymph nodes and bone or, in fewer instances, to the brain. It is the metastases that produce the poorer clinical outcomes of the disease. With regard to prostate cancer metastases, steps along the pathway to malignant transformation must first occur to produce the life-threatening cell types. It is likely that all metastatic cells should share some features in common despite differences in tissue specificities in their spread. If a protein structure could be identified that has a relationship to this common metastatic pathway, its targeting could provide a manner by which to treat currently incurable metastatic disease.

We propose that surface vimentin could be such a common marker of highly metastatic cancer cells and as well possibly related to prostate cancer stem- or progenitor cells. Proteome analysis indicated vimentin expression correlated with invasion and metastases of androgen-independent prostate cancers [4]. To achieve a long-lasting cure in cancer therapy, it is envisioned that cancer stem cells must also be eliminated as well [5]. Vimentin has been previously linked to the metastatic potential of cancer cells as its increased expression has been demonstrated to be a marker of epithelial-mesenchymal transition (EMT) in prostate cancer [6]. Prostate cancer cells must undergo EMT for invasiveness and metastases to occur. Critical alterations that occur during EMT of primary epithelial tumor cells result in tumor cells capable of penetrating the extracellular matrix and accessing lymphatic and blood vessels for tumor metastases.

Interestingly, EMT is a normal process that occurs in embryonic development during organogenesis allowing epithelial cells to differentiate into spindle-shaped cells with mesenchymal properties showing or possessing invasive properties. The developing embryonic organ invasion fronts resemble fronts of tumor metastases, and cells undergoing EMT could share characteristics of primitive tissue stem cells and actually represent tumor stem cell populations. A link between EMT and hypoxia may be a trigger of prostate cancer metastases in the tumor microenvironment. Vimentin expression was induced under hypoxic conditions and corresponded to increases in invasive metastatic potential of LNCaP tumor cells [7]. Cell surface expression of the SC5 mAb domain of vimentin was observed for cutaneous T-cell lymphomas [8] and earlier the CLNH11.4 MAb was shown to detect vimentin on the surface of a variety of malignant cells including prostate cancer cells, but not on healthy cells [9-11]. With regard to therapeutic efficacy and targeting, glioma patients showed reduced tumor growth after intravenous treatments with the vimentin-specific antibody CLN-IgG [12].

The prostate cancer cell lines DU145, LNCaP and PC3 can be passaged and used to form xenograft tumors. Prostate cancer cells have previously been reported to be heterogeneous in their tumorigenic capacity [13]. In accordance with the cancer stem cell models that posit only a rare subset of cells are tumor-initiating cells, several markers for prostate cancer stem cells have been reported including the cell surface markers CD44 and CD133 [14,15]. In a previous report, stem-like cells isolated from prostate cancer patient tumors were shown to express both CD44 and CD133 and this population had the highest colony forming capacity and could differentiate into multiple cell types [16]. CD44 sorted tumor cell populations have been reported to be enriched for tumorigenic and metastatic progenitor cells [17]. There is now considerable evidence that CD44 expression is linked to cancer-initiation cells (CIC) and stem- or progenitor cells [18]. CD133 is another marker associated with being part of CICs and interestingly with regard to targeting has been shown to be located on the same membrane microdomain as the CD44 molecule [19]. Previously, a CD44+α2β1+CD133+ population of cells isolated from the DU145 prostate cancer line was shown to have the capacity for self-renewal and reported to be a marker of prostate cancer stem cells [20]. Here, we examined the co-expression of surface vimentin with the CD44 and CD133 stem- or progenitor cell marker proteins.

We demonstrate that two different domains of vimentin are detectable on the surface for each of three commonly studied prostate cancer cell lines LNCaP, PC­3 and DU145. Each of these cell lines was established from metastases isolated from different lesion sites in the body corresponding to lymph node, bone and brain. For each tumor line, both SC5 and V9 mAbs specific for vimentin were capable of detecting surface expression representing two different regions of the protein. In addition, we found major populations of cells that express vimentin with CD44 and only a minor subset that express these molecules with CD133, a potential marker on cancer stem cells. Finally, we demonstrate that Cowpea mosaic virus (CPMV) nanoparticles are capable of binding to the surface of both LNCaP and PC3 cells. CPMV nanoparticles have been extensively studied have for potential applications in nanomedicine. CPMV nanoparticles are monodisperse and about 30 nm in size; they can be engineered with imaging moieties and therapeutic molecules using bioconjugate chemistry [21]. The *in vivo* properties of CPMV are well understood; after intravenous inoculation CPMV particles are cleared rapidly from circulation with no apparent toxicity or pathological effects [22]. CPMV nanoparticles were previously shown to bind surface vimentin on other human tumor cells [22,23]. While we have not obtained a rigorous experimental proof that vimentin binding extrapolates to the prostate cancer cells, we suggest vimentin domains are recognized by CPMV. These results provide the possibility of creating therapeutic agents capable of targeting vimentin in combination with other surface markers to prevent cancer metastases as well as kill cancer stem cells.

## 2. Materials and Methods

### 2.1. Cell Culture

The prostate cancer cell lines DU145, LNCaP and PC3 were obtained from American Type Culture Collection (Manassas, VA, USA). Dulbecco's modified Eagle's medium (DMEM) was used to culture the cell lines, supplemented with 10% fetal bovine serum (FBS), 100 μg/mL streptomycin and 100 IU/mL of penicillin.

### 2.2. Immunofluorescence and Flow Cytometric Analysis

For fluorescence microscopy, SC5 ascites [24] and V9 mAb (Sigma, St. Louis, MO, USA) were used for experiments to detect surface and cytoplasmic vimentin expression. For SC5 mAb staining, the goat anti-mouse IgM FITC conjugate (Invitrogen) was used as the 2º detection mAb and as a control alone. For V9, a goat anti-mouse Alexa Flour 488 was used as the 2º detection mAb and as a control alone. In this analysis, a total of 5 × 10^4^ prostate cancer cell lines or HMEC-1 cells were grown on 35 mm glass bottom petri dishes (Matek, Ashland, MA, USA) overnight at 37 ºC in 2 mL of medium. For surface staining, cells were fixed with 3% paraformaldehyde, 0.3% gluteraldehyde, 1mM MgCl_2_ in PBS pH 7.2 for 5 min at RT. The cells were then blocked for 45 min with 5% PBS at RT followed by staining with vimentin antibody SC5 or V9 (1:200 in PBS) and then incubated for 45 minutes at 4 ºC. For cytosolic detection, an additional permeabilization step was included following fixation using 0.2% Triton X-100 in PBS for 2 min at RT. Cell nuclei were stained using 4′,6-diamidino-2-phenylindole (DAPI) (1:1000 for 5 min at room temperature). Three washings in PBS were performed between each step of the staining procedure. Dishes were imaged using a Biorad 2100 confocal microscope.For flow cytometric analysis, prostate cancer cells were displaced by treatment with 1× Citric Saline instead of trypsinization to prevent any potential proteolytic cleavage of surface vimentin molecules that had been previously reported [9]. The SC5 anti-vimentin ascites was used in this analysis along with CD44-PE (eBioscience) and CD133-APC (Catalog# 130-090-826, Miltenyi Biotec) mAbs as well as isotype controls. Goat anti-mouse IgM FITC mAb was used as the negative control for vimentin stainings. Cells were collected in 96-well U-bottom shaped plates in 100 μL portions at a concentration of 5 × 10^6^ cells/mL. Prior to staining, cells were fixed for 5 min at RT. To stain for cell surface vimentin non-permeabilized cells were stained with SC5 ascites at a dilution of 1:200 and incubated at 4 °C for 45 min in FACS buffer (PBS pH 7.4 containing 1 mM EDTA, 25 mM HEPES and 1% FBS). The secondary goat anti-mouse IgM-FITC conjugated antibody was then used at a 1:200 dilution in FACS buffer and incubated for 45 min at 4 °C. For counter-staining experiments, cells were then incubated with CD44-PE and subsequently CD133-APC mAb with 3 washes in PBS between each incubation step. Analysis was performed on a FACSCalibur flow cytometer (Becton Dickinson, Mountain View, CA, USA).

### 2.3. CPMV Uptake by Confocal Microscopy

CPMV was propagated in Vigna unguiculata and purified using established procedures [25]. PEG2000, Oregon Green 488 (O488), and/or Alexa Fluor 647 (A647) were covalently attached to surface Lys residues on CPMV using N-hydroxysuccinimide (NHS) activated esters. PEG2000-NHS (NANOCS, New York, NY, USA), O488-NHS (*6 isomer*), or A647-NHS (Invitrogen, Carlsbad, CA, USA) was dissolved in DMSO and added to CPMV in a molar excess of 3000 to CPMV (2–3 mg mL^−1^), and the reaction was carried out for 2 hours or overnight at room temperature in a PBS pH 7.2:DMSO mixture of 8:2. Samples were purified using gradient ultracentrifugation in 10–40% sucrose gradients in 0.1 M phosphate buffer pH 7.0 (Beckman SW 28 Ti rotor, 28700 rpm, 3 hours, 4 °C) followed by ultra-pelleting (Beckman 50.2 Ti rotor, 42000 rpm, 3 hours, 4 °C).

For CPMV uptake analysis, cells were grown as described above. And then 5 μg CPMV-O488 particles were added in growth media and cells were incubated at 37 **°**C and 5% CO2 for 3 hours. Excess CPMV was removed and cells incubated for additional 3 hours in growth medium, prior to fixation, blocking, staining of the nuclei, mounting, and imaging as described above. In additional experiments, cell membranes were also stained using Alexa Fluor 555-labeled wheat germ agglutinin (WGA-A555) provides an assessment based on visualization for internalization of CPMV nanoparticles.

## 3. Results

### 3.1. Expression of two Different Domains of Vimentin as Detected by SC5 and V9 mAbs on the Surface of DU145, LNCaP and PC3 Prostate Cancer Cell Lines

**Figure 1.**
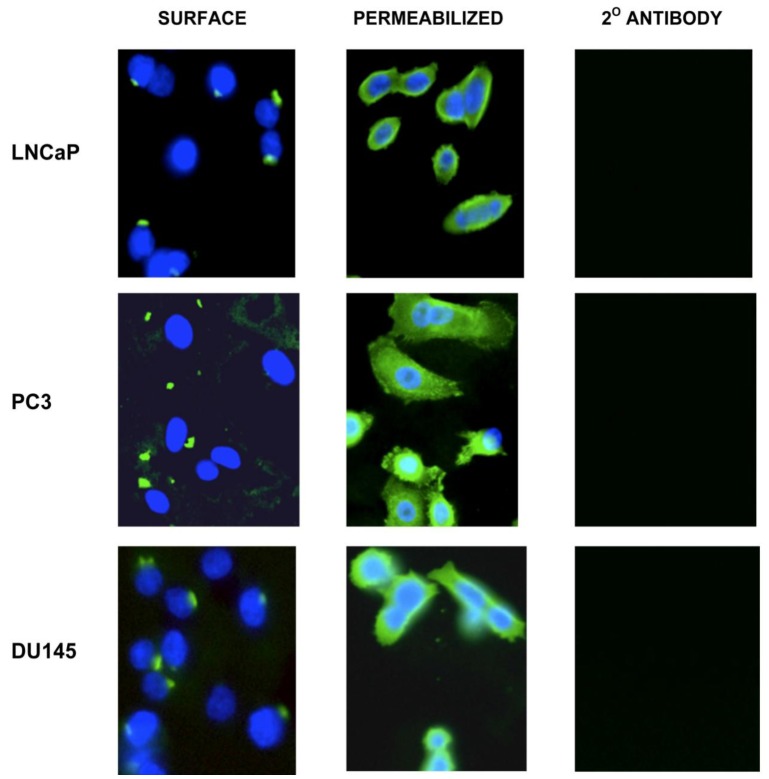
Vimentin is detected on the surface of DU145, LNCaP and PC3 tumor cells by SC5 mAb on non-premeabilized cells by confocal microscopy (40×). Intracellular expression is also detected in cells permeabilized by treatment with Triton-X 100. The goat anti-mouse FITC 2° mAb used alone as a control produced no staining pattern. Similar staining results were obtained with the anti-vimentin mAb V9 (data not shown). Results indicate two different epitope domains of vimentin are present on the surface of each prostate cancer cell line.

The DU145, LNCaP and PC3 cell lines were isolated from three distinct metastatic sites from prostate cancer patients from brain, lymph node and bone, respectively. We determined whether at least two different domains of vimentin could be displayed on the surface of these metastatic lines as dual targeting of closely associated epitopes could be utilized for possible improvements of targeted immunotherapy. As shown in Figure 1, differential expression of surface *versus* cytosolic vimentin was detectable for each of these metastatic cell lines following confocal microscopy analysis. Using SC5 mAb recognizing vimentin, a punctate staining pattern on the surface of each cell line was detected for DU145, LNCaP and PC3 cells. It is not clear why a punctate staining pattern occurs or why not all cells stain positive for vimentin. In addition, the intensity of DU145 vimentin cell surface staining appears less intense in comparison to PC3 and LNCaP cells. It is possible that detection of surface vimentin is dependent on a density threshold and that the absence of staining could reflect different stages of cell differentiation or assemblage of hemidesmosomes. This polarized (punctate) localization pattern of surface vimentin has been previously reported on HeLa, HT-29 and HuT-78 cells [8,22,23]

The vimentin staining pattern was contrastingly different following permeabilization of the cells with Triton-X 100. Here, each of the permeabilized cell lines demonstrated a more diffuse and intense staining pattern of the cytoplasmic compartment.whereas cells stained with the secondary goat anti-mouse control antibody produced no staining pattern. SC5 is a monoclonal antibody that recognizes the rod 1 coil domain of the vimentin protein. In data not shown, the V9 mAb produced an identical staining pattern similar to that seen for the SC5 antibody with punctate surface and diffuse cytoplasmic staining. V9 recognizes a distinct carboxy terminal domain of vimentin. Interestingly as seen from the figure, not all cells had detectable surface vimentin expression; this is consistent with what was found for surface vimentin expression on cervical, breast and colon cancer cell lines [23]. Thus, these results demonstrate that at least two unique domains of vimentin are expressed on the surface of each of the metastases derived prostate tumor cell lines.

### 3.2. Flow Cytometric Examination Reveals Differential Expression of Surface Vimentin between the Three Different Prostate Metastatic Cancer Cell Lines

**Figure 2.**
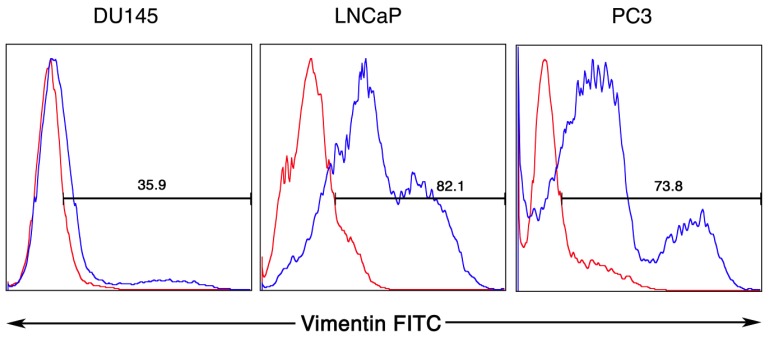
Characterization of vimentin detected on the surface of DU145, LNCaP and PC3 prostate cancer cell lines. Flow cytometry analyses were performed on cells that were displaced with 1× Citric Saline so as not to proteolytically disturb surface molecule expression. Cell staining was performed with SC5 mAb followed by FITC-conjugated 2º mAb.

Earlier examination of PC3 cells revealed a hierarchy in subpopulations of cells in culture for their tumorigenic capacity with holoclones expressing CD44 being stem-like cells with tumor initiating activity [26]. To begin to examine for differences in function of vimentin positive cells, we first performed flow cytometry analysis using the SC5 mAb (Figure 2). We were able to detect both qualitative and quantitative differences in the surface expression of vimentin between the three different prostate cancer lines. DU145 cells revealed a much lower percentage of positive staining for surface vimentin expression in comparison to both the LNCaP and PC3 cell lines: 35.9% of DU145 cells stained for surface vimentin in comparison to 82.1% for LNCaP and 73.8% of PC3 cells, respectively. Vimentin expression appeared to be dichotomous with high and intermediate staining populations of cells for each tumor type.

### 3.3. Flow Cytometric Examination Reveals Robust Surface Expression of CD44 on all three of the Metastatic Prostate Cancer Cell Lines

**Figure 3.**
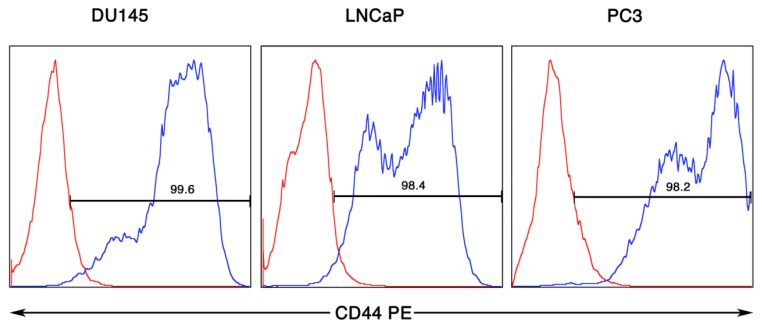
Characterization of CD44 detected on the surface of DU145, LNCaP and PC3 prostate cancer cell lines. Flow cytometry analyses were performed on cells that were displaced with 1X Citric Saline so as not to proteolytically disturb surface molecule expression. Cell staining was performed with CD44-PE conjugated mAb.

CD44 is an important surface receptor protein that binds hyaluronan and is involved in cell adhesion and migration [27]. With regard to prostate cancer progression, CD44 has been reported to be on the population of tumor cells enriched in tumorigenic potential [17]. We therefore, examined the metastatic tumor cell lines for their surface expression of CD44. As shown in Figure 3, each of the three different metastatic prostate tumor cell lines examined had remarkably high percentages of CD44 positively staining cells with each line staining for CD44 at greater than 95%. This high percentage of staining detected could be related to the citric saline procedure for detachment of the cells. There is a stalk-like structure between the N-terminus globular domain and the transmembrane domain that contains many putative proteolytic cleavage sites [28]. CD44 expression also appeared to be dichotomous as reflected by the presence of high and intermediate staining populations within the prostate cancer cell lines. Cells used in these experiments were all derived from freshly split cells and used within 24 hours for analysis. It is unclear if this could also impact the detection of CD44 in our hands. Regardless from our analysis, the majority of cells from each metatstatic tumor line express the CD44 molecule on their surface.

### 3.4. Flow Cytometric Examination Reveals Vimentin and CD44 Are Co-expressed with CD133 in a Small Subpopulation for all three of the Metastasis-Derived Prostate Cancer Cell Lines

**Figure 4.**
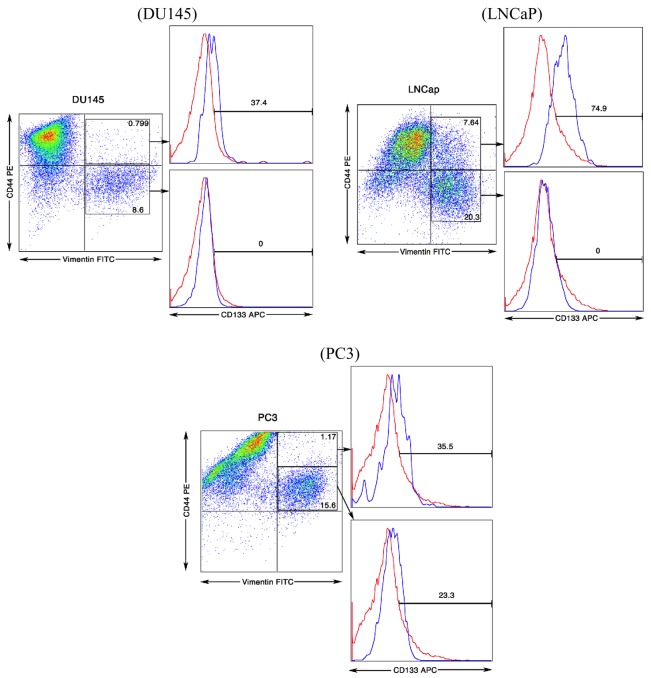
Characterization of surface vimentin+ CD44+ CD133+ population of cells on DU145, LNCaP and PC3 prostate cancer cell lines. Flow cytometry analysis was performed on cells that were displaced with 1X Citric Saline so as not to proteolytically disturb surface molecule expression. Cell staining was performed with SC5 mAb directed against vimentin, CD44-PE and CD133-APC mAbs.

A CD44+α2β1CD133+ population of cells was previously characterized as a stem cell in primary and metastasized prostate cancers [13,16]. We next determined whether surface vimentin would co-express with CD44 and CD133. As shown in Figure 4, co-expression of surface vimentin with the CD44 and CD133 molecules was found on a small subpopulation of cells. This surface expression pattern of vimentin could mark stem- or progenitor-like cells in all three metastatic prostate lines if CD133 and CD44 are also co-expressed in this population. Interestingly, CD133 expression appeared to preferentially mark CD44 high populations of cells. As a population, Vimentin+CD44+CD133+ cells represented approximately only 0.3, 0.4 and 5% of the total sorted tumor cell population for the DU145, PC3 and LNCaP lines, respectively. PC3 cells have been characterized to have a higher metastatic capacity *versus* DU145 followed by LNCaP cells that are not invasive [29,30]. Given the expression profile of these three cell types, CD133 expression would not appear to associate with invasive capacity. However, there does appear to be some correlation with the intensity of CD44 expression and metastastic invasiness. The intensity of CD44 expression for PC3 cells qualitatively shifted almost half a log higher compared to both DU145 and LNCaP cells. Clearly additional factors must contribute, as LNCaP cells are the least invasive of the cell types. CD44 has been shown to associate with membrane-associated metalloprotease that cleaves CD44 to produce a modified ectodomain that can enhance tumor cell migration [31]. It is interesting to speculate that this cleavage could account for better binding to CD133 epitope 1 recognized on PC3 cells by this mAb but this concept remains to be tested. Analysis of CD44 and vimentin expression revealed two major populations of cells (data not shown). We found one population of cells to be Vimentin-High, CD44-Intermediate and another to be Vimentin-Intermediate, CD44-High. It is intriguing to speculate that these two populations actually represent meroclones or paraclones and that the CD44-High, Vimentin-High are holoclones that harbor stem-like cancer cells that has been reported for PC3 cells in culture [26].

### 3.5. Vimentin Is not Detected on the Surface of HMEC-1 Cells a Surrogate Cell Type for Vascular Endothelium

**Figure 5.**
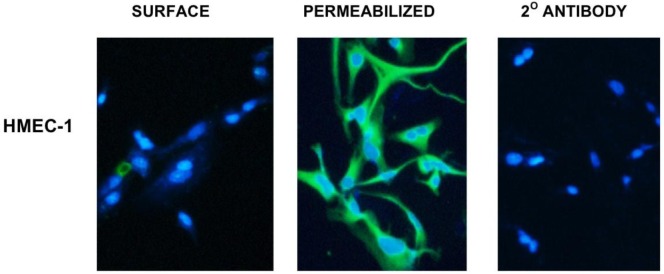
Vimentin is not detected on the surface of HMEC-1 cells by SC5 mAb on non-premeabilized cells by confocal microscopy (40X). Intracellular expression is detected upon permeabilization by treatment with Triton-X 100. The goat anti-mouse FITC 2º mAb used alone as a control, produced no staining pattern. Results indicate that the SC5 domain of vimentin is not present on the surface of human microvascular endothelial cells.

The HMEC-1 cell line was generated by transfection with human telomerase catalytic protein to prevent unwanted activities that SV40 transduction might contribute to cellular function [32]. HMEC-1 cells were derived from primary human microvascular endothelial cells and serve as an *in vitro* model for human endothelium. As an approximation of *in vivo* vascular binding capacity, we examined HMEC-1 cells against SC5 mAb to determine if surface vimentin is present (Figure 5). We found that unlike the metastatic prostate cancer lines, no surface vimentin is detectable on HMEC-1 cells. However, permeabilized cells still produced the diffuse cytoplasmic vimentin detection consistent with its role as an intermediate filament protein. This result suggests that intravenous delivery might not inhibit vimentin-targeted delivery of tumor therapeutic agents either through antibody or nanoparticles if targeted through an appropriate vimentin domain. It has previsouly been shown that anti-vimentin antibodies can be specifically targeted to tumors *in vivo*. Data indicate that surface vimentin is preferentially expressed on tumor endothelium compared to healthy vasculature [33]. This is consitent with previous reports showing that CPMV is preferentially localized to tumor endothelium [34]. Furthermore, it was shown that CPMV nanoparticles could be targeted to surface vimentin-expressing HT-29 tumors *in vivo* [23]. The expression, display, function, and availability of surface vimentin or its CPMV binding epitope(s) are unknown. It remains to be determined what forms of vimentin are surface displayed at sites of tumors, its neovasculature *versus* other possible tissue target sites that could complicate the delivery of therapeutic agents.

### 3.6. Cowpea Mosaic Virus (CPMV) Nanoparticles Are Capable of Targeting PC3 and LNCaP Prostate Tumor Cells through Surface Vimentin

**Figure 6.**
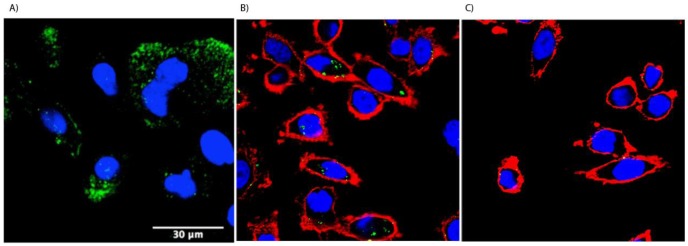
5 × 10^4^ cells were grown in 35 mm glass bottom petri dishes (Matek, Ashland, MA, USA) overnight at 37 °C and 5% CO2. For CPMV binding and uptake studies, (**A**) 1 μg CPMV-O488 particles (1 × 10^6^ particles per cell) were added in growth media and cells were incubated at 37 °C and 5% CO2 for 3 hours, prior to fixing using 4% paraformaldehyde and 0.3% glutaraldehyde in PBS pH 7.2 for 5 min at room temperature. Cell nuclei were stained by adding 4′,6-diamidino-2-phenylindole (DAPI) (1:9500 for 10 min at room temperature). Slides were mounted using Vecta Shield mounting medium (Vector Laboratories, Burlingame, CA, USA). (**B**+**C**) cell membranes were stained using Alexa Fluor 555-labeled wheat germ agglutinin (WGA-A555) and CPMV uptake could be demonstrated (**B**), PEGylated CPMV particles do not interact with the cells (**C**). Imaging was performed using a Biorad 2100 confocal microscope with a 60x oil objective. Data were analyzed and images were created using ImageJ.

We next determined whether the metastatic prostate tumor cells could be targeted with a nanoparticle, CPMV. CPMV nanoparticles can be functionalized with a variety of moieties used in imaging in therapy (reviewed in [21]). CPMV were previously shown to bind to surface vimentin on cells [22] and these nanoparticles were capable of localizing to HT-29 tumors (a highly aggressive human colon carcinoma line) *in vivo* using the chick chorioallantoic membrane experimental human tumor xenograft tumor model [23]. As shown in Figure 6A, PC3 cells are shown to be capable by binding to 1 μg CPMV-O488 particles (1 × 10^6^ particles per cell) after only 3 hours of incubation. A similar binding staining pattern was found for the LNCaP cell line (data not shown). In Figure 6B, cell membranes on PC3 cells were stained using Alexa Fluor 555-labeled wheat germ agglutinin (WGA-A555). Internalization of CPMV nanoparticles is suggested by the appearance of the green staining within the red membrane WGA-A555 fluorescence marker. However, more rigorous proof of internalization is still required as is the specific requirement of vimentin expression for the prostate cancer lines. To confirm specificity, CPMV particles were conjugated to polyethylene glycol (PEG). PEGylation is an effective strategy to reduce biospecific interactions. We recently demonstrated that CPMV particles covalently attached to PEG2000 (termed P2 formulation) were effectively shielded and vimentin-mediated cell interactions are significantly blocked up to 3 hours [35]. Such control experiments were conducted, and uptake of P2 particles in PC3 cells was not apparent (data not shown). These data further indicate vimentin-specific targeting of CPMV to surface vimentin-positive prostate cancer cells. However, testing with known inhibitors for internalization, vimentin competition or blocking antibody studies remain to be done. It is likely that surface vimentin can be targeted on metastatic prostate cancer cells by this nanoparticle. LNCaP cells did not appear to internalize the CPMV nanoparticles (data not shown). We do not know what accounts for this difference between the cell lines as it is currently unknown what domain(s) of vimentin are required for CPMV interaction or internalization.

## 4. Discussion

In this report, we demonstrate for the first time that multiple epitope domains of vimentin are expressed on the surface of three different prostate cancer cell lines derived from three different metastatic tissue sites. It is a highly desirable goal to be able to target metastases. Thus, the detection by SC5 and V9 mAbs recognizing the rod 1 coil and C-terminus region of vimentin on the surface of these metastatic cells could provide unique targets in the development of novel therapeutic agents designed to eliminate metastases. Moreover, we were able to demonstrate that surface vimentin is co-expressed on the surface of three different metastasis-derived prostate cancer cell lines with CD44 and CD133 molecules. Several studies have indicated CD44+ populations of cells to be enriched in tumorigenic stem cells [17,36]. Preparative enrichment and transfer of this cell population enriched in CD44 and CD133 have been done in the past to demonstrate progenitor potential associated with this population of cells. It is interesting to speculate that these triple expressing Vimentin+CD44+CD133+ phenotype of cells are CSCs. However, these studies remain to be carried out for the Vimentin+CD44+CD133+ prostate cancer cells

What role CD44 might have in the hierarchy of stem cell function could be related to its association with membrane-type 1 matrix metalloproteinase (MT1-MMP). CD44 has been shown to direct MT1-MMP to front of migrating cell lamellipodia [37]. MT1-MMP is required for degradation of the extracellular matrix for cancer cell migration. From our analysis, differences in the intensity of CD44 expression were detected between the PC3, LNCaP and DU145 cell lines. An increased intensity in CD44 staining on the PC3 cell line was detected in comparison the DU145 and LNCaP cell lines. However, it is likely that this increased signaling cannot account totally for differences in invasiveness as LNCaP cells stained qualitatively similar to that of the slightly more invasive DU145 cell line. It is intriguing to speculate that proteolytic processing of CD44 accounts for any additional invasiveness of the tumor lines. This aspect of function remains further to be explored.

CD133 is a transmembrane pentaspan protein that is described as a surface marker on human hematopoietic cells [38]. CD133 alone or in combination has been used as identification of cancer stem cell populations from metastatic tumors of brain [39], liver [40], pancreas [41], lung [42], colon [43] and the prostate [16]. However, CD133 expression has been reported to not solely be restricted to tumor initiating cells [44] and therefore finding additional molecule markers that may identify cancer stem cells is important. We were unable to directly detect any major shifts in CD133 on the bulk population of DU145, LNCaP and PC3 cells by flow cytometry analysis with the CD133 mAb (data not shown). However when CD133 populations were analyzed in CD44 by surface vimentin sorting, we found a subpopulation of cells for each tumor cell type that expressed all three surface markers. This population would be consistent with being a prostate cancer stem cell. Experiments are underway to examine whether this subpopulation has the ability for self-renewal.

With regard to prostate cancer targeting, we were able to show uptake of CPMV nanoparticles by the prostate cancer cell lines and that HMEC-1 cells (surrogate cells for human vascular endothelium) did not bind the SC5 vimentin specific mAb. This result indicates that intravenous delivery of vimentin targeting antibody constructs or nanoparticles may not be taken up non-specifically in the vasculature and preferential target metastatic tumor cells. Indeed previous studies have shown that CPMV homing to tumors *in vivo* is observed [23,34]. Furthermore peptide targeted and native CPMV particles were shown to localize to PC3 tumors *in vivo* [Steinmetz 2011 Prostate Imaging, Small]. We suggest the potential utility of CPMV nanoparticle to target prostate cancers via vimentin interactions, this, however, requires formal testing. An additional possible benefit for targeting through vimentin recognition is that a unique extracellular deposition for vimentin has now been shown to mark the stroma of prostate cancer lesions but not prostate hyperplasia or normal prostate epithelial tissue [45]. This unique stromal distribution pattern could also aide and serve as highly selective target for novel therapeutic agents against this cancer. In addition, increased expression of vimentin has also been found to localize on tumor neovasculature and this expression could also aide in the targeting or serve as a target in vimentin-directed cancer agents.

We found that surface vimentin is detectable on the metastases-derived prostate cancer cells. This finding is consistent with other studies on expression of vimentin for metastatic *versus* non-metastatic breast cancer cells [23]. Surface vimentin expression may be a common marker for various metastatic cancers. Thus, targeted delivery of engineered therapeutic CPMV nanoparticles may open a strategy for treatment of metastatic disease.

Finally, determining the actual topography of what domains of vimentin are expressed on the surface of prostate and other metastatic cancer cells could provide insights for the creation of novel therapeutic antibody agents, such as spectral-targeted antibody derivative constructs [46]. These agents are capable of binding multiple antigen targets simultaneously. Therefore, domains of vimentin that are displayed on the surface as well as the co-expressed CD44 and/or CD133 molecules could be used in therapeutic targeting. Such dual binding constructs could provide better tumor specificity through the added avidity of binding of each domain. The characterization of surface vimentin domain topography is of interest but still remains to be fully determined to utilize for targeted therapy.

## 5. Conclusions

We describe in this report that at least two different epitope domains of vimentin can be detected on the surface of three different prostate cancer tumor cell lines derived from different metastatic tissue sites. This commonality in expression on the metastatic lines makes surface vimentin an interesting and possibly universal target by therapeutic agents designed to treat metastases. Surface vimentin was found to be co-expressed on a subpopulation of cells along with CD44 and CD133 suggested to be markers of prostate cancer stem cells. Whether surface vimentin also marks stem- or pro-genitor cells, remains to be formally demonstrated. Finally as a proof-in-principle, we were able to demonstrate CPMV nanoparticles can target prostate cancer cells. Our findings suggest the possibility of creating novel nanoparticle or antibody derivative constructs using vimentin as a way to target prostate cancer metastases and/or stem cells therapeutically.
